# Genetic Analysis of *mlh3* Mutations Reveals Interactions Between Crossover Promoting Factors During Meiosis in Baker’s Yeast

**DOI:** 10.1534/g3.112.004622

**Published:** 2013-01-01

**Authors:** Megan Sonntag Brown, Elisha Lim, Cheng Chen, K. T. Nishant, Eric Alani

**Affiliations:** * Department of Molecular Biology and Genetics, Cornell University, Ithaca, New York 14853-2703; † School of Biology, Indian Institute of Science Education and Research, Thiruvananthapuram, India 695016

**Keywords:** DNA mismatch repair, meiotic recombination, Msh4-Msh5, Mlh1-Mlh3, crossing over

## Abstract

Crossing over between homologous chromosomes occurs during the prophase of meiosis I and is critical for chromosome segregation. In baker’s yeast, two heterodimeric complexes, Msh4-Msh5 and Mlh1-Mlh3, act in meiosis to promote interference-dependent crossing over. Mlh1-Mlh3 also plays a role in DNA mismatch repair (MMR) by interacting with Msh2-Msh3 to repair insertion and deletion mutations. Mlh3 contains an ATP-binding domain that is highly conserved among MLH proteins. To explore roles for Mlh3 in meiosis and MMR, we performed a structure−function analysis of eight *mlh3* ATPase mutants. In contrast to previous work, our data suggest that ATP hydrolysis by both Mlh1 and Mlh3 is important for both meiotic and MMR functions. In meiotic assays, these mutants showed a roughly linear relationship between spore viability and genetic map distance. To further understand the relationship between crossing over and meiotic viability, we analyzed crossing over on four chromosomes of varying lengths in *mlh3Δ mms4Δ* strains and observed strong decreases (6- to 17-fold) in crossing over in all intervals. Curiously, *mlh3Δ mms4Δ* double mutants displayed spore viability levels that were greater than observed in *mms4Δ* strains that show modest defects in crossing over. The viability in double mutants also appeared greater than would be expected for strains that show such severe defects in crossing over. Together, these observations provide insights for how Mlh1-Mlh3 acts in crossover resolution and MMR and for how chromosome segregation in Meiosis I can occur in the absence of crossing over.

During gametogenesis in most eukaryotes, crossing over between homologous chromosomes occurs during prophase of meiosis I and is critical for both chromosome segregation and exchange of genetic information between homologs (Zickler 2006). Meiotic recombination in *Saccharomyces cerevisiae* is initiated by the induction of approximately 140−170 *SPO11*-dependent double-strand breaks (DSBs) that are located throughout the genome (Cao *et al.* 1990; Gilbertson and Stahl 1996; Keeney *et al.* 1997; Robine *et al.* 2007; Chen *et al.* 2008). Roughly 40% of these DSBs are repaired to form crossovers between homologous chromosomes; the rest are repaired as noncrossovers or by using a sister chromatid as template. DSB resection results in 3′ single-strand tails whose repair is directed primarily to the complementary sequence in the other homolog (Schwacha and Kleckner 1995). The 3′ tails are acted upon by strand exchange enzymes to form single-end invasion intermediates (SEIs). SEIs are subsequently converted into double Holliday junctions (dHJs) that are ultimately resolved into crossovers (Hunter and Kleckner 2001).

Two MutS and MutL homolog (MSH and MLH) complexes, Msh4-Msh5 and Mlh1-Mlh3, respectively, promote crossovers that are nonrandomly spaced (interference-dependent crossover pathway). In this pathway the presence of one crossover decreases the likelihood of another nearby (Kleckner *et al.* 2004; Stahl *et al.* 2004; Shinohara *et al.* 2008). A second, interference-independent crossover pathway is mediated by the endonuclease complex Mus81-Mms4 (Clyne *et al.* 2003; De Los Santos *et al.* 2003; Argueso *et al.* 2004; Matos *et al.* 2011). Little is known about the intermediates in this pathway; however, the Mus81-Mms4 complex is thought to act directly in Holliday junction resolution or by cleaving D-loops and half-HJ intermediates (Kaliraman *et al.* 2001; Hollingsworth and Brill 2004; Gaskell *et al.* 2007). Genetic, biochemical, and physical studies have shown that Msh4-Msh5 acts in meiosis to stabilize SEI and dHJ intermediates (Börner *et al.* 2004; Snowden *et al.* 2004; Nishant *et al.* 2010). Mlh3 was found to coimmunoprecipitate with Msh4, suggesting that the Mlh1-Mlh3 heterodimer interacts with the Msh4-Msh5-DNA complex (Santucci-Darmanin *et al.* 2002). This interaction is thought to reinforce the crossover decision by providing a substrate for a dHJ resolvase(s) during early- to mid-pachytene stages in meiosis (Wang *et al.* 1999; Santucci-Darmanin *et al.* 2002; Hoffman and Borts 2004; Whitby 2005; Nishant *et al.* 2008). Consistent with these observations are cytological observations showing that ∼140 Msh4-Msh5 foci are present per mouse spermatocyte nucleus in zygotene. The number of Msh4 foci decrease to about two to three foci per chromosome in mid-pachytene. At this stage, Mlh1 foci begin to appear. Initially, there is high (95–100%) colocalization between the two foci; however, as pachytene progresses, this colocalization gradually disappears (Kneitz *et al.* 2000; Santucci-Darmanin *et al.* 2000; Svetlanov and Cohen 2004). The presence of a large number of Msh4-Msh5 foci in zygotene supports early roles for Msh4-Msh5 in meiosis, perhaps during initial interhomolog interactions (Storlazzi *et al.* 2010).

Crossover placement in meiosis is carefully regulated through the Msh4-Msh5 interference pathway and the actions of Sgs1 helicase, which may play a role in promoting crossing over, as well as serve as an anticrossover factor by removing aberrant recombination intermediates (Jessop *et al.* 2006; Oh *et al.* 2007; De Muyt *et al.* 2012; Zakharyevich *et al.* 2012). Crossover levels also are regulated by a homeostasis mechanism that ensures that when DSB levels are reduced crossovers are maintained at the expense of noncrossovers. This mechanism facilitates proper disjunction of homologs (Martini *et al.* 2006; Zanders and Alani 2009). At least one crossover per homolog, called the obligate crossover, appears necessary for proper homolog disjunction. Steps that ensure the obligate crossover in the interference-dependent pathway are thought to occur during the crossover/noncrossover decision step, just before single-end invasion (Allers and Lichten 2001; Hunter and Kleckner 2001).

During DNA mismatch repair (MMR), the MSH proteins Msh2-Msh6 and Msh2-Msh3 bind to base−base and insertion/deletion mismatches that form primarily as the result of DNA replication errors (Kunkel and Erie 2005). In the baker’s yeast *S. cerevisiae* Msh2-Msh6 and Msh2-Msh3 interact primarily with a single MLH complex, Mlh1-Pms1, to reinforce the repair decision and activate downstream excision and resynthesis steps. In addition to its role in meiosis outlined previously, Mlh1-Mlh3 performs a minor role in the repair of insertion and deletions, most likely through interactions with Msh2-Msh3 (Flores-Rozas and Kolodner 1998). Mlh3 contains an ATP-binding domain that is highly conserved among MLH proteins. It also contains an endonuclease domain that is detected in specific classes of MLH proteins [Figure 1 (Kadyrov *et al.* 2006)]. Previous work from our laboratory indicated that the endonuclease domain present near the C-terminus of Mlh3 is critical for its role in MMR and meiotic crossing over (Nishant *et al.* 2008).

**Figure 1:**
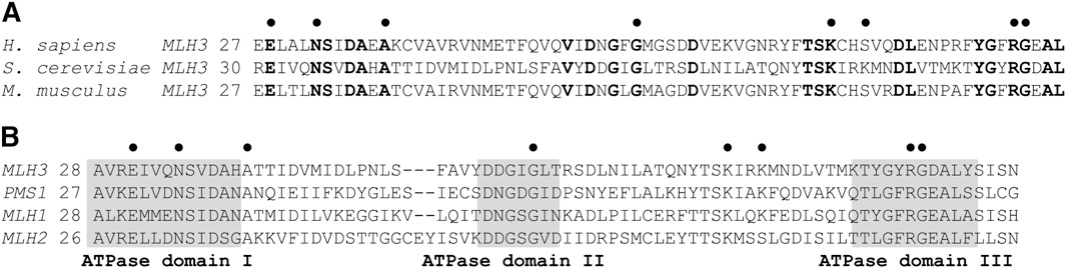
The ATPase domain of Mlh3 is highly conserved across eukaryotic species and within the MLH protein family. (A) Location of the *mlh3* mutations analyzed in this study with respect to *Homo sapiens*, *S. cerevisiae*, and *Mus musculus* protein sequences. Conserved residues are highlighted in bold. (B) Location of the *mlh3* mutations created with respect to the conserved ATPase domains in the *Saccharomyces cerevisiae* MLH family of proteins (Ban and Yang 1998; Tran and Liskay 2000). ATPase domain IV is not shown.•, locations of *mlh3* alleles analyzed in this study.

In this study we investigated the role of Mlh3 in DNA MMR and meiosis by analyzing the phenotype of eight *mlh3* ATPase mutants. Our data suggest that ATP hydrolysis by both Mlh1 and Mlh3 is important for both meiotic and MMR functions. In meiotic assays these mutants showed a roughly linear relationship between spore viability and genetic map distance. To further analyze the role of Mlh3 in meiosis, we analyzed crossing over on four chromosomes in *mlh3Δ mms4Δ* cells and observed a strong decrease in crossing over at all intervals, but higher spore viability than would be expected for strains that show such strong crossover defects. Together these observations provide insights for how Mlh1-Mlh3 acts in crossover resolution and MMR, and for how chromosome segregation in Meiosis I can occur in the absence of crossing over.

## Materials and Methods

### Media

*S. cerevisiae* strains were grown at 30° in either yeast extract-peptone, 2% dextrose media, or minimal selective media (SC) containing 2% dextrose, sucrose, or galactose (Rose *et al.* 1990). When required for selection, geneticin (Invitrogen, San Diego, CA) and nourseothricin (Werner BioAgents, Jena, Germany) were used at recommended concentrations (Wach *et al.* 1994; Goldstein and McCusker 1999). Sporulation plates and media were prepared as described in Argueso *et al.* (2004).

### Plasmids and strains

Plasmids containing each of the *mlh3* alleles were constructed via QuickChange mutagenesis (Stratagene, La Jolla, CA) using the single-step integration vector pEAI254 as a template. pEAI254 contains the SK1 *MLH3* gene with a *KANMX4* selectable marker inserted 40 bp downstream of the stop codon (Nishant *et al.* 2008). Mutations created by QuickChange were confirmed by sequencing (Sanger method) the entire *MLH3* open reading frame. Primer sequences used to create the *mlh3* alleles are available upon request. pEAI254 and mutant derivatives were digested with *Bam*HI and *Sal*I before introduction into yeast by the lithium acetate transformation method (Gietz *et al.* 1995). Plasmids used for the dominant-negative assay were constructed by QuickChange mutagenesis using pEAE220 (S288C, *GAL10-MLH3*, *2μ*, *URA3)* as a template (Nishant *et al.* 2008). The mutated regions created by QuickChange were subcloned into a new pEAE220 backbone to eliminate other possible mutations.

**Figure 2:**
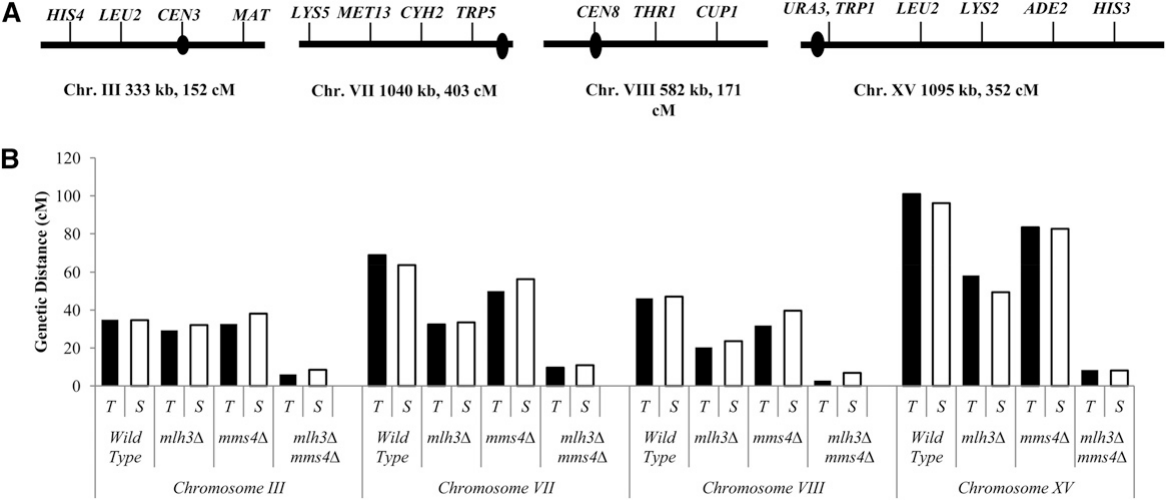
Cumulative genetic distances for wild type, *mlh3Δ*, *mms4Δ*, and *mlh3Δ mms4Δ* on four chromosomes. (A) Location of genetic markers used to determine map distances in the NHY942/NHY943 background for chromosomes III, VII, VIII, and the EAY1108/EAY1112 background for chromosome XV. (B) The cumulative genetic distance for each chromosome is shown for both complete tetrad data (black bars) and single spore data (white bars). Raw data are shown in Table 7. Data for wild type for chromosomes III, VII, and VIII are from Zanders and Alani (2009). Data for wild type and *mms4Δ* for chromosome XV are from Argueso *et al.* (2004). Data for *mlh3Δ* and *mlh3Δ mms4Δ* on chromosome XV are from Nishant *et al.* (2008). For chromosome III, the physical distances (end of the marker gene to the beginning of the next, in KB) are: *HIS4-LEU2*, 23; *LEU2-CEN3*, 22; *CEN3-MAT*, 90. For chromosome VII, the physical distances are: *LYS5-MET13*, 56, *MET13-CYH2*, 36; *CYH2-TRP5*, 135. For chromosome VIII, the physical distances are: *CEN8-THR1*, 54; *THR1-CUP1*, 52. For chromosome XV, the physical distances are: *URA3-LEU2*, 136; *LEU2-LYS2*, 43; *LYS2-ADE2*, 59; *ADE2-HIS3*, 157.

The SK1 *mlh3* alleles described in this study were introduced by gene replacement into SK1 congenic and isogenic strain backgrounds (Tables 1 and 2). The effect of the eight alleles on spore viability and crossing over was measured in EAY1108/1112 [SK1 congenic; Figure 2 (Argueso *et al.* 2004)]. *mlh3msh5* double mutants also were constructed in EAY1108/1112. More specifically, *mlh3* alleles were introduced by gene replacement into the *msh5Δ MATα* strain EAY1279, and *msh5* alleles were introduced into the *mlh3Δ msh5Δ MATa* strain EAY3312. The *mlh3Δ* and *mlh3Δ mms4Δ* strains analyzed in Figure 2 were derived from the SK1 isogenic NHY942/NHY943 background (De Los Santos *et al.* 2003).

**Table 1:** Yeast strains used in this study

Strain	Genotype
EAY1062	*MATa ho*::*hisG*, *ura3*, *leu2*::*hisG*, *ade2*::*LK*, *his4xB*, *lys214*::*insE-A14*
EAY2186	*MATa ho*::*hisG*, *ura3*, *leu2*::*hisG*, *ade2*::*LK*, *his4xB*, *lys214*::*insE-A14*, *MLH3*::*KANMX4*
EAY2037	*MATa ho*::*hisG*, *ura3*, *leu2*::*hisG*, *ade2*::*LK*, *his4xB*, *lys214*::*insE-A14*, *mlh3*::*KANMX4*
EAY3117	*MATa ho*::*hisG*, *ura3*, *leu2*::*hisG*, *ade2*::*LK*, *his4xB*, *lys214*::*insE-A14*, *mlh3-E31A*::*KANMX4*
EAY3119	*MATa ho*::*hisG*, *ura3*, *leu2*::*hisG*, *ade2*::*LK*, *his4xB*, *lys214*::*insE-A14*, *mlh3-N35A*::*KANMX4*
EAY3121	*MATa ho*::*hisG*, *ura3*, *leu2*::*hisG*, *ade2*::*LK*, *his4xB*, *lys214*::*insE-A14*, *mlh3-A41F*::*KANMX4*
EAY3123	*MATa ho*::*hisG*, *ura3*, *leu2*::*hisG*, *ade2*::*LK*, *his4xB*, *lys214*::*insE-A14*, *mlh3-G63R*::*KANMX4*
EAY3125	*MATa ho*::*hisG*, *ura3*, *leu2*::*hisG*, *ade2*::*LK*, *his4xB*, *lys214*::*insE-A14*, *mlh3-K80E*::*KANMX4*
EAY3127	*MATa ho*::*hisG*, *ura3*, *leu2*::*hisG*, *ade2*::*LK*, *his4xB*, *lys214*::*insE-A14*, *mlh3-K83A*::*KANMX4*
EAY3129	*MATa ho*::*hisG*, *ura3*, *leu2*::*hisG*, *ade2*::*LK*, *his4xB*, *lys214*::*insE-A14*, *mlh3-R96A*::*KANMX4*
EAY3131	*MATa ho*::*hisG*, *ura3*, *leu2*::*hisG*, *ade2*::*LK*, *his4xB*, *lys214*::*insE-A14*, *mlh3-G97A*::*KANMX4*
EAY1269	*MATa ura3*, *leu2*, *trp1*, *lys2*::*insE-A14*
EAY1366	*MATa leu2*, *ura3*, *trp1*, *his3*, *lys2*::*insE-A14 mlh1Δ*::*KANMX4*
EAY3308	*MATa ura3*, *leu2*, *trp1*, *lys2*::*insE-A14 w/ pEAE220 (GAL10-MLH3*, *2μ)*
EAY3309	*MATa ura3*, *leu2*, *trp1*, *lys2*::*insE-A14 w/ pEAE374 (GAL10-mlh3-E31A*, *2μ)*
EAY3310	*MATa ura3*, *leu2*, *trp1*, *lys2*::*insE-A14 w/ pEAE375 (GAL10-mlh3-R96A*, *2μ)*
EAY3311	*MATa ura3*, *leu2*, *trp1*, *lys2*::*insE-A14 w/ pEAE376 (GAL10-mlh3-G97A*, *2μ)*
EAY1108	*MATa trp1:hisG leu2*::*hisG ho*::*hisG ura3 lys2 URA3insertion@CENXV LEU2insertion@chromXV*, *LYS2 insertion at position 505193*
EAY2413	Same as EAY1108, but *mlh3Δ*::*NATMX4*
EAY3007	Same as EAY1108, but *mlh3-E31A*
EAY3009	Same as EAY1108, but *mlh3-N35A*
EAY3011	Same as EAY1108, but *mlh3-A41F*
EAY3013	Same as EAY1108, but *mlh3-G63R*
EAY3015	Same as EAY1108, but *mlh3-K80E*
EAY3017	Same as EAY1108, but *mlh3-K83A*
EAY3019	Same as EAY1108, but *mlh3-R96A*
EAY3021	Same as EAY1108, but *mlh3-G97A*
EAY2423	Same as EAY1108, but *msh5-D76A*::*KANMX4*
EAY2439	Same as EAY1108, but *msh5- T423A*::*KANMX4*
EAY2032	Same as EAY1108, but *mlh3Δ*::*KANMX4*, *msh5Δ*::*NATMX4*
EAY1281	Same as EAY1108, but *msh5Δ*::*NATMX4*
EAY1847	Same as EAY1108, but *mlh3Δ*::*KANMX4*
EAY1845	Same as EAY1108, but *mms4Δ*::*NATMX4*
EAY2030	Same as EAY1108, but *mlh3Δ*::*KANMX4*, *mms4Δ*::*NATMX4*
EAY3312	Same as EAY1108, but *mlh3Δ*::*HPHMX4*, *msh5Δ*::*NATMX4*
EAY3313	Same as EAY1108, but *mlh3Δ*::*HPHMX4*, *msh5-D76A*::*KANMX4*
EAY3314	Same as EAY1108, but *mlh3Δ*::*HPHMX4*, *msh5-T423A*::*KANMX4*
EAY1112	*MATα ura3*, *trp1*::*hisG*, *leu2*::*hisG*, *lys2*, *ho*::*hisG*, *ade2*::*hisG*, *his3Δ*::*hisG*, *TRP1insertion@CENXV*
EAY1848	Same as EAY1112, but *mlh3Δ*::*KANMX4*
EAY1846	Same as EAY1112, but *mms4Δ*::*NATMX4*
EAY1279	Same as EAY1112, but *msh5Δ*::*NATMX4*
EAY2031	Same as EAY1112, but *mlh3Δ*::*KANMX4*, *mms4Δ*::*NATMX4*
EAY2033	Same as EAY1112, but *mlh3Δ*::*KANMX4*, *msh5Δ*::*NATMX4*
EAY3315	Same as EAY1112, but *mlh3-R96A*::*KANMX4*, *msh5Δ*::*NATMX4*
EAY3316	Same as EAY1112, but *mlh3-G97A*::*KANMX4*, *msh5Δ*::*NATMX4*
EAY1425/NHY942	*MATα ho*::*hisG ade2Δ can1 ura3(ΔSma-Pst) met13-B trp5-S CENVIII*::*URA3 thr1-A cup1s*
EAY2904	Same as EAY1425, but *mlh3Δ*::*KANMX4*
EAY3290	Same as EAY1425, but *mms4Δ*::*KANMX4*
EAY3296	Same as EAY1425, but *mlh3Δ*::*KANMX4 mms4Δ*::*KANMX4*
EAY1426/NHY943	*MATa ho*::*hisG ade2Δ ura3(ΔSma-Pst) leu2*::*hisG CENIII*::*ADE2 lys5-P his4-B cyh2*
EAY2906	Same as EAY1426, but *mlh3Δ*::*KANMX4*
EAY3323	Same as EAY1426, but *mms4Δ*::*NATMX4*
EAY3298	Same as EAY1426, but *mlh3Δ*::*KANMX4 mms4Δ*::*NATMX4*

**Table 2:** Diploids generated by the zero growth mating regime that were analyzed for spore viability and genetic map distance

EAY1108/EAY1112 Background (Analyzed in Tables 4, and 5 and Figures 2, 3, and 4)	
EAY1108/EAY1112	*wild type*
EAY1108/EAY1848	*MLH3/mlh3Δ*
EAY2413/EAY1848	*mlh3Δ/mlh3Δ*
EAY3007/EAY1848	*mlh3-E31A/mlh3Δ*
EAY3009/EAY1848	*mlh3-N35A/mlh3Δ*
EAY3011/EAY1848	*mlh3-A41F/mlh3Δ*
EAY3013/EAY1848	*mlh3-G63R/mlh3Δ*
EAY3015/EAY1848	*mlh3-K80E/mlh3Δ*
EAY3017/EAY1848	*mlh3-K83A/mlh3Δ*
EAY3019/EAY1848	*mlh3-R96A/mlh3Δ*
EAY3021/EAY1848	*mlh3-G97A/mlh3Δ*
EAY1281/EAY1279	*msh5Δ/msh5Δ*
EAY2032/EAY2033	*msh5Δ mlh3Δ/msh5Δ mlh3Δ*
EAY2423/EAY1279	*msh5-D76A/msh5Δ*
EAY2439/EAY1279	*msh5-T423A/msh5Δ*
EAY3313/EAY3315	*msh5-D76A mlh3G96A/msh5Δ mlh3Δ*
EAY3313/EAY3316	*msh5-D76A mlh3-G97A/msh5Δ mlh3Δ*
EAY3314/EAY3315	*msh5-T423A mlh3-R96A/msh5Δ mlh3Δ*
EAY3314/EAY3316	*msh5-T423A mlh3-G97A/msh5Δ mlh3Δ*
EAY1845/EAY1846	*mms4Δ/mms4Δ*
EAY2030/EAY2031	*mlh3Δ mms4Δ/mlh3Δ mms4Δ*
NHY942/NHY943 background (analyzed in Tables 6, 7, 8, Figure 2)	
NHY942/NHY943	*wild type*
EAY2904/EAY2906	*mlh3Δ/mlh3Δ*
EAY3290/EAY3323	*mms4Δ/mms4Δ*
EAY3296/EAY3298	*mlh3Δ mms4Δ/mlh3Δ mms4Δ*

The indicated haploid strains (Table 1, *Materials and Methods*) were mated and sporulated using the zero growth mating protocol and tetrads were dissected (Argueso *et al.* 2003).

The isogenic SK1 strain EAY1062 [*lys2*::*InsE-A_14_* (Nishant *et al.* 2008)] was used to measure the effect of *mlh3* mutations on mutation rate (Table 3). For the dominant-negative assay, pEAE220 (2μ, S288c *GAL10-MLH3*), and mutant derivatives pEAE374 (*GAL10-mlh3-E31A*), pEAE375 (*GAL10-mlh3-R96A*), and pEAE376 (*GAL10-mlh3-G97A*) were transformed into EAY1269 (S288c, *lys*::*InsE-A_14_*).

**Table 3:** Reversion of the *lys2:InsE-A_14_* allele in *mlh3* strains

Genotype	n	Mutation Rate (×10^−7^)	Relative to WT	Phenotype
*MLH3*	110	4.71 (3.87–5.11)	1.0	+
*mlh3Δ*	110	26.5 (23.5–30.4)	5.7	−
*mlh3-E31A*	15	30.5 (16.7–51.6)	6.5	−
*mlh3-N35A*	15	31.2 (25.6–44.4)	6.7	−
*mlh3-A41F*	15	27.9 (17.1–34.3)	6.0	−
*mlh3-G63R*	15	23.8 (18.2–37.1)	5.1	−
*mlh3-K80E*	15	16.0 (15.1–27.7)	3.4	−
*mlh3-K83A*	15	5.24 (3.49–6.34)	1.1	+
*mlh3-R96A*	15	14.8 (6.42–40.6)	3.2	−
*mlh3-G97A*	15	16.6 (11.8–26.0)	3.6	−
*MLH3* + empty vector	11	4.42 (1.02-6.05)	1	+
*MLH3* + *pGAL10-MLH3*	11	39,100 (15,700-79,900)	8850	−
*MLH3* + *pGAL10-mlh3E31A*	11	47,800 (28,700-85,900)	10,800	−
*MLH3* + *pGAL10-mlh3R96A*	11	23,500 (5910-38,400)	5320	−
*MLH3* + *pGAL10-mlh3G97A*	11	96,000 (45,800-156,000)	21,700	−
*mlh1Δ +* empty vector	11	218,000 (121,000-283,000)	49,300	−

The *lys2:InsE-A_14_* SK1 strain EAY1062 and *mlh3* derivatives (Table 1) were examined for reversion to Lys^+^. EAY1269 (*lys2:InsE-A_14_*, S288c strain) and an *mlh1Δ* derivative containing the indicated overexpression plasmids were tested for reversion to Lys^+^. n, the number of independent cultures tested from at least two independently constructed strains. Median mutation rates are presented with 95% confidence intervals, and relative mutation rates compared with the wild-type strain are shown. WT, wild type.

### Genetic map distance analysis

EAY1108/EAY1112 and NHY942/NHY943 background diploids were sporulated using the zero growth mating protocol [Table 2 (Argueso *et al.* 2003)] and tetrads were dissected. For the EAY1108/EAY1112 background strains, tetrads were dissected and spores were germinated on synthetic complete media. For the NHY942/NHY943 background strains, tetrads were dissected and germinated on yeast extract-peptone, 2% dextrose media supplemented with complete amino acids. Spore clones were incubated 3–4 d at 30° and then replica-plated to various selective media. The replica plates were scored after 1 d of incubation at 30°. Spore clones were analyzed using the recombination analysis software RANA (Argueso *et al.* 2004), which analyzes map distances. Genetic map distances ± SE were calculated using the Stahl Laboratory Online Tools (http://www.molbio.uoregon.edu/∼fstahl/), which uses the formula of Perkins (1949). Differences in spore formation and viability were analyzed by a χ^2^ test with *P*-values < 0.05 considered statistically significant. The genetic intervals measured in this study (illustrated in Figure 2) were: chromosome III-*HIS4-LEU2*, *LEU2-CEN3*, *CEN3-MAT*; chromosome VII-*LYS5-MET13*, *MET13-CYH2*, *CYH2-TRP5*; chromosome VIII-*CEN8-THR1*, *THR1-CUP1*; and chromosome XV- *URA3-LEU2*, *LEU2-LYS2*, *LYS2-ADE2*, *ADE2-HIS3*.

### Lys^+^ reversion assays

The *mlh3* allele constructs were transformed into EAY2037 (SK1, *mlh3Δ*::*KANMX4*, *lys2*::*InsE-A_14_*), and strains were analyzed for reversion to Lys^+^ (Tran *et al.* 1997). At least 15 independent cultures for each allele were analyzed, and experiments were conducted with two independent transformants. Mutation rates were determined as previously described (Drake 1991; Heck *et al.* 2006). Each median rate was normalized to the wild-type median rate to calculate the fold-increase in mutation rate. 95% confidence intervals were determined as described (Dixon and Massey 1969).

For the dominant-negative assays, EAY1269 bearing pEAE220 and mutant derivatives were grown for 5 d on uracil dropout SC agar plates containing 2% sucrose or 2% sucrose and 2% galactose. Individual colonies were picked and grown overnight in liquid (-agar) versions of the respective media for 26 hr. Appropriate dilutions were made, and cells grown in sucrose only were plated on uracil, lysine dropout SC agar plates containing 2% sucrose, and uracil dropout SC agar plates containing 2% glucose. Cells grown in sucrose and galactose were plated on uracil, lysine dropout SC agar plates containing 2% sucrose and 2% galactose, and uracil dropout SC agar plates containing 2% glucose. Using *GAL10-MLH3* and *mlh1Δ* as controls, we analyzed 11 independent colonies from two independent transformations.

## Results and Discussion

### ATP hydrolysis by both Mlh1 and Mlh3 is likely to be important for their roles in meiosis and MMR

MLH family proteins each contain an N-terminal ATP binding domain. This domain is thought to regulate asymmetric conformational changes in MLH dimers through cycles of ATP binding and hydrolysis (Ban and Yang 1998; Ban *et al.* 1999; Tran and Liskay 2000; Hall *et al.* 2002; Sacho *et al.* 2008). Previous structure−function studies have shown that the two subunits in yeast Mlh1-Pms1 are functionally asymmetric. For example, the Mlh1 subunit of the yeast Mlh1-Pms1 complex displayed a much greater affinity for ATP compared to the Pms1 subunit, and an ATP hydrolysis mutation in *MLH1* (*mlh1-E31A*) conferred a much greater effect on MMR than the equivalent mutation in *PMS1* (*pms1-E61A*; Tran and Liskay 2000; Hall *et al.* 2002). Also, in baker’s yeast the Mlh1 subunit has been shown to interact with the downstream MMR factor Exo1 in an ATP-dependent manner. Thus, ATP-dependent and asymmetric conformational changes in MLH proteins are likely to be important to modulate interactions with downstream MMR effector molecules (Pedrazzi *et al.* 2001; Tran *et al.* 2001).

Previous genetic and biochemical analyses identified mutations in the ATP-binding domains of yeast MLH proteins that disrupt ATP hydrolysis to a greater extent than ATP binding (*e.g.*, *mlh1-E31A*). Mutations also were identified that severely disrupt ATP binding [*e.g.*, *mlh1-N35A* (Hall *et al.* 2002)]. Other mutations have been made in MLH ATP-binding domains that are predicted to affect ATP binding and/or ATP-dependent conformational changes but have yet to be tested in biochemical assays [Figure 1 (Tran and Liskay 2000; Hall *et al.* 2002; Ban and Yang 1998; Ban *et al.* 1999)].

We made mutations in Mlh3 predicted to confer defects in ATP hydrolysis (*mlh3-E31A*) and binding *(mlh3-N35A*), and six other mutations that map within or near motifs identified in the GHKL family of ATPases, of which the MLH proteins are members [Figure 1 (Ban and Yang 1998; Ban *et al.* 1999)]. We tested the effect of these mutations in a MMR repair assay that measures reversion of the *lys2*::*InsE-A_14_* allele (Tran *et al.* 1997) and in meiotic assays that measure spore viability and crossing over in four intervals on chromosome XV in EAY1108/1112 SK1 congenic strains [Figure 2 (Argueso *et al.* 2004)]. Three of the eight *mlh3* mutations also were analyzed by Cotton *et al.* (2010), using similar assays. In the *lys2*::*InsE-A_14_* reversion assay, *mlh3Δ* strains display a roughly 6-fold increase in mutation rate compared with wild-type (Harfe *et al.* 2000; Nishant *et al.* 2008; this study). We found that all but one of the eight *mlh3* alleles conferred MMR defects similar to the null (within 95% confidence intervals), ranging from 3.2 to 6.7-fold greater than wild-type levels. *mlh3-K83A* strains showed a wild-type phenotype (Table 3). Our results for the *mlh3-N35A* and *mlh3-G97A* mutations were similar to those obtained by Cotton *et al.* (2010). However, for *mlh3-E31A*, which is thought to disrupt ATP hydrolysis by the Mlh3 subunit, we observed a null MMR phenotype; Cotton *et al.* (2010) observed a close to wild-type phenotype for this mutant.

To assess Mlh3 expression, we overexpressed *mlh3-E31A*, *mlh3-R96A*, and *mlh3-G97A* in wild-type cells and assessed dominant-negative phenotypes using the *lys2*::*InsE-A14* frameshift reporter, which can detect a roughly four-order of magnitude difference in mutation rate (Tran *et al.* 1997). This approach was taken because we have been unable to detect single copy levels of Mlh3 in vegetative cells (M. Rogacheva and E. Alani, unpublished observations). We showed previously that overexpressing Mlh3 using the *GAL10* promoter conferred a high mutator phenotype in the *lys2*::*InsE-A_14_*, reversion assay with mutation rates more than 1000-fold greater than wild-type. This phenotype was similar to that seen in wild-type strains overexpressing Mlh1 (Shcherbakova and Kunkel 1999; Nishant *et al.* 2008). Based on these observations, we hypothesized that increased levels of Mlh3 interfered with mismatch repair by outcompeting Pms1 for Mlh1, thus preventing Mlh1-Pms1 from acting in MMR (Wang *et al.* 1999; Kondo *et al.* 2001). Consistent with this idea, overexpressing mlh3-E529K, which does not interact with Mlh1, did not confer a dominant-negative phenotype (Nishant *et al.* 2008). As shown in Table 3, each allele conferred a strong dominant-negative phenotype similar to *MLH3*, with mutation rates 5000- to 20,000-fold greater than wild-type containing an empty vector. This suggests that an intact Mlh1-mlh3 complex is formed in each of these mutants.

As mentioned previously, mismatch repair rates have been examined in strains bearing *mlh1* mutations at positions equivalent to those made in *MLH3* (Tran and Liskay 2000; Argueso *et al.* 2003; Hoffman *et al.* 2003; Wanat *et al.* 2007). Consistent with its lesser role in MMR, *mlh3* alleles show a lower mutation rate as measured in the *lys*::*InsE-A_14_* reversion assay compared with equivalent *mlh1* alleles; however, they appear to be just as sensitive to mutagenesis. Similar to their equivalent *mlh3* mutations, *mlh1-K81E*, *mlh1-R97A*, and *mlh1-G98A* conferred null phenotypes in MMR. *mlh1-E31A* and *mlh1-K84A*, however, conferred MMR phenotypes that were different from their equivalent *mlh3* mutations, with *mlh1-E31A* strains appearing more proficient in MMR and *mlh1-K84A* strains less proficient [Tables 3 and 4 (Tran and Liskay 2000; Hoffman *et al.* 2003; Wanat *et al.* 2007; Argueso *et al.* 2003)]. Thus our work, in conjunction with previous studies, reinforces the hypothesis that the subunits of MLH complexes provide unique contributions to MMR (Tran and Liskay 2000; Hall *et al.* 2002; Argueso *et al.* 2003; Hoffman *et al.* 2003; Wanat *et al.* 2007; Nishant *et al.* 2008; Cotton *et al.* 2010).

**Table 4:** Spore viabilities, map distances, qualitative MMR phenotypes, and known *mlh1* homolog phenotypes for the *mlh3* alleles, *msh5Δ*, and *mlh3 msh5* double mutants

Strain	Spore Viability, %	cM	MMR	*mlh1 allele*	MMR
*mlh3* mutant analysis					
*MLH3**^a^*	97.0	100.9 (1068)	+	*MLH1*	+
*mlh3Δ**^b^*	71.7	54.5 (582)	−	*mlh1Δ*	−
*mlh3-E31A*	89.2	67.0 (330)	−	*mlh1-E31A**^c^*,*^d^*	+/−
*mlh3-N35A*	72.7	51.5 (229)	−	*mlh1-E35A*	ND
*mlh3-A41F*	71.6	51.2 (214)	−	*mlh1-A41F*	ND
*mlh3-G63R*	74.1	51.2 (216)	−	*mlh1-G64R*	ND
*mlh3-K80E*	71.8	49.8 (221)	−	*mlh1-K81E**^e^*	−
*mlh3-K83A*	94.1	100.5 (289)	+	*mlh1-K84A**^d^*	+/−
*mlh3-R96A*	82.4	76.4 (177)	−	*mlh1-R97A**^d^*	−
*mlh3-G97A*	81.5	61.0 (210)	−	*mlh1-G98A**^c^*,*^f^*	−
*msh5* mutant analysis					
*msh5Δ**^a^*	36.0	37.0 (540)			
*msh5Δ mlh3Δ*	31.8	38.5 (43)			
*msh5-D76A**^g^*	87.8	53.9 (77)			
*msh5-T423A**^g^*	95.2	78.3 (101)			
*msh5-D76A mlh3 R96A*	57.8	45.0 (81)			
*msh5-D76A mlh3 G97A*	47.1	31.7 (82)			
*msh5-T423A mlh3 R96A*	89.6	60.9 (160)			
*msh5-T423A mlh3 G97A*	78.3	54.7 (130)			

Spore viabilities (%) and cumulative genetic map distances from four spore-viable tetrads (number in parentheses) on chromosome XV are shown for wild-type, *mlh3*, and *msh5* strains in the SK1 congenic EAY1108/1112 background (Table 2). The qualitative MMR phenotype of each allele (see Table 3) is shown for comparison. MMR data are also shown for the homologous *mlh1* alleles, if known. MMR, mismatch repair; ND, not determined.

a Data obtained from Argueso *et al.* (2004).

b Data obtained from Nishant *et al.* (2008).

c Data from Tran and Liskay (2000).

d Data from Argueso *et al.* (2003).

e Data from Wanat *et al.* (2007).

f Data from Hoffman *et al.* (2003).

g Data obtained from Nishant *et al.* (2010).

We tested the effect of *mlh3* mutations in meiosis in the EAY1108/1112 SK1 congenic strain background, which is marked to measure map distances over four consecutive genetic intervals on chromosome XV [*Materials and Methods*; Figure 2 (Argueso *et al.* 2004)]. In this background, wild-type display 97% spore viability and a cumulative map distance of 100.9 cM over the four intervals, whereas *mlh3Δ* display 72% spore viability and a cumulative map distance of 54.5 cM (Tables 4 and 5). As shown in Tables 4 and 5, four of eight of the *mlh3* mutations (*mlh3-N35A*, *-A41F*, *G63R*, *K80E*) conferred null phenotypes in the meiotic assays, and one mutation, *mlh3-K83A*, conferred a wild-type phenotype. Three mutations, *mlh3-E31A*, *mlh3-R96A*, and *mlh3-G97A*, conferred intermediate phenotypes (Tables 4 and 5). Like Cotton *et al.* (2010), we found that the predicted ATP binding mutation *mlh3-N35A* conferred a null phenotype in the meiotic assays. However, in contrast to a nearly wild-type phenotype previously seen for *mlh3-E31A* in both MMR and meiotic assays (Cotton *et al.* 2010), we found that *mlh3-E31A* mutants displayed, compared with the wild-type, defects in meiosis (Table 4; 67 cM map distance, 89% spore viability, *P* < 0.001) and MMR (null phenotype, Table 3). Thus, our analyses are consistent with ATP hydrolysis by Mlh3 being important for its meiotic and MMR functions. We do not have a clear explanation for why our data differ from Cotton *et al.* (2010). However, one possibility is that the SK1 strain background is more sensitized to defects in *MLH3* compared with the Y55 background studied by Cotton *et al.* (2010). Consistent with this idea, we found that SK1 *mlh3Δ* strains showed lower spore viability (72%) compared with Y55 *mlh3Δ* strains [92% (Cotton *et al.* 2010)].

**Table 5:** Genetic map distances for chromosome XV from single spores and tetrads with distributions of parental and recombinant progeny

	Single Spores				Tetrads				
Genotype	n	Par.	Rec	cM	n	PD	TT	NPD	cM
*URA3-LEU2*									
Wild type*^a^*	4644	3635	1009	21.7	1068	607	456	5	21.8-23.8
* msh5Δ**^a^*	5674	5352	322	5.7	757	643	76	1	5.0-6.4
* mlh3Δ**^b^*	3023	2682	341	11.3	582	460	114	8	12.3-15.5
* msh5Δ mlh3Δ*	382	352	30	7.9	43	34	8	0	6.5-12.6
* msh5-D76A**^c^*	351	310	41	11.7	77	57	17	0	9.0-13.9
* msh5-T423A**^c^*	457	378	79	17.3	101	62	33	0	14.9-19.8
* mlh3- R96A*	840	676	164	19.5	177	105	69	0	18.0-21.7
* mlh3- G97A*	978	841	137	14.0	210	152	55	2	13.6-18.5
* msh5-D76A mlh3 R96A*	462	409	53	11.5	81	63	16	0	7.9-12.4
* msh5-D76A mlh3 G97A*	490	455	35	7.1	82	71	11	0	4.8-8.6
* msh5-T423A mlh3 R96A*	717	583	134	18.7	160	96	64	0	18.1-21.9
* msh5-T423A mlh3 G97A*	622	552	70	11.3	130	100	28	1	10.3-16.1
*LEU2-LYS2*									
Wild type*^a^*	4644	3388	1256	27.0	1068	496	569	3	26.6-28.4
* msh5Δ**^a^*	5674	5047	627	11.1	757	562	155	3	11.0-13.0
* mlh3Δ**^b^*	3023	2610	413	13.7	582	424	154	3	12.9-16.6
* msh5Δ mlh3Δ*	382	338	44	11.5	43	31	10	1	11.5-26.6
* msh5-D76A**^c^*	351	308	43	12.3	77	58	16	0	8.4-13.2
* msh5-T423A**^c^*	457	365	92	20.1	101	57	38	0	17.5-22.5
* mlh3- R96A*	840	695	145	17.3	177	112	62	0	16.0-19.6
* mlh3- G97A*	978	825	153	15.6	210	140	68	1	15.6-19.8
* msh5-D76A mlh3 R96A*	462	422	40	8.7	81	67	12	0	5.6-9.6
* msh5-D76A mlh3 G97A*	490	457	33	6.7	82	72	10	0	4.3-7.9
* msh5-T423A mlh3 R96A*	717	606	111	15.5	160	111	49	0	13.5-17.1
* msh5-T423A mlh3 G97A*	622	535	87	14.0	130	91	37	1	13.7-19.6
*LYS2-ADE2*									
Wild type*^a^*	4644	4052	592	12.7	1068	803	263	2	12.1-13.7
* msh5Δ**^a^*	5674	5409	265	4.7	757	659	61	0	3.7-4.7
* mlh3Δ*^b^	3023	2822	201	6.6	582	501	81	0	6.2-7.7
* msh5Δ mlh3Δ*	382	363	19	5.0	43	39	3	0	1.6-5.6
* msh5-D76A**^c^*	351	320	31	8.8	77	60	14	0	7.2-11.7
* msh5-T423A**^c^*	457	405	52	11.4	101	75	20	0	8.4-12.6
* mlh3- R96A*	840	775	65	7.7	177	149	25	0	5.9-8.5
* mlh3- G97A*	978	898	80	8.2	210	173	35	1	7.9-11.7
* msh5-D76A mlh3 R96A*	462	437	25	5.4	81	68	11	0	5.0-8.9
* msh5-D76A mlh3 G97A*	490	464	26	5.3	82	75	7	0	2.7-5.8
* msh5-T423A mlh3 R96A*	717	669	48	6.7	160	141	19	0	4.7-7.2
* msh5-T423A mlh3 G97A*	622	591	31	5.0	130	116	13	0	3.7-6.4
*ADE2-HIS3*									
Wild type*^a^*	4644	3033	1611	34.7	1068	343	709	16	36.5-38.9
* msh5Δ**^a^*	5674	4797	877	15.5	757	496	215	9	17.2-20.2
* mlh3Δ**^b^*	3023	2485	538	17.8	582	379	201	2	17.1-19.5
* msh5Δ mlh3Δ*	382	328	54	14.1	43	30	12	0	10.8-17.8
* msh5-D76A**^c^*	351	277	74	21.1	77	43	31	0	18.1-23.8
* msh5-T423A**^c^*	457	322	135	29.5	101	44	49	2	27.4-36.9
* mlh3- R96A*	840	600	240	28.6	177	74	98	2	28.7-34.5
* mlh3- G97A*	978	801	177	18.1	210	136	73	0	15.8-19.1
* msh5-D76A mlh3 R96A*	462	395	67	14.5	81	57	20	2	14.6-25.9
* msh5-D76A mlh3 G97A*	490	422	68	13.9	82	58	24	0	12.1-17.1
* msh5-T423A mlh3 R96A*	717	575	142	19.8	160	97	63	0	17.8-21.6
* msh5-T423A mlh3 G97A*	622	507	115	18.5	130	83	45	1	16.8-22.8

Strains used are isogenic derivatives of the congenic SK1 EAY1108/1112 background (Tables 1 and 2). Single spore data are shown with n, total number of spores, and parental and recombinant data. Map distances (cM) were calculated by recombination frequency (recombinant spores/total spores) × 100. Tetrad data are shown with n, number of complete tetrads. Map distances (cM) were calculated using the Perkins formula (Perkins 1949), and 95% confidence intervals were calculated using the Stahl Laboratory Online Tools website (http://www.molbio.uoregon.edu/∼fstahl/).

a Data from Argueso *et al.* (2004).

b Data from Nishant *et al.* (2008).

c Data from Nishant *et al.* (2010).

It is important to note that five of the eight *mlh3* alleles displayed consistent phenotypes in both the MMR and meiosis assays (either wild-type or null in both). However, three *mlh3* hypomorph mutants, *mlh3-E31A*, *-R96A*, *-G97A*, displayed null phenotypes in MMR, but intermediate meiotic phenotypes, as measured in meiotic spore viability and crossover assays (Tables 4 and 5). These observations suggest that, as was seen for Mlh1 (Argueso *et al.* 2003; Hoffman *et al.* 2003), Mlh3 functions are more easily disrupted for MMR.

### *mlh3* strains show a roughly linear relationship between crossing over and spore viability

**Figure 3:**
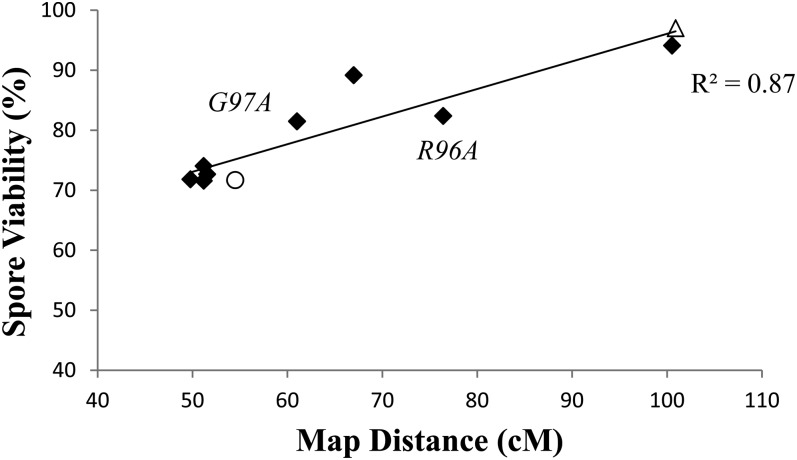
*mlh3* strains show a roughly linear relationship between crossing over and spore viability. Spore viabilities are plotted *vs.* genetic map distances on chromosome XV for eight *mlh3* ATP binding domain mutations, wild type (open triangle), and *mlh3Δ* (open circle).

As shown in Figure 3 and Table 4, the *mlh3* mutants displayed a relationship where spore viability decreased progressively with map distance (R^2^ = 0.87). Consistent with this we observed that wild-type spore viability was significantly greater than that seen in *mlh3-E31A*, *-R96A*, and *-G97A* (*P* ≪ 0.001). This pattern is in contrast to the pattern observed in *msh4/5* mutants, where crossing over could be decreased to approximately 50% of wild-type levels (to ∼50 cM across the four intervals in chromosome XV) without an apparent defect in spore viability, after which point spore viability and crossing over decreased linearly (Nishant *et al.* 2010). Based on this and other observations, Nishant *et al.* (2010) proposed that crossover designation functions executed by Msh4-Msh5 are prioritized in yeast to maintain the obligate crossover, ensuring that each homolog pair receives at least one disjunction-promoting crossover. The finding that *mlh3* mutants show a pattern where spore viability decreased progressively with map distance is consistent with a wealth of data supporting a crossover resolution role for Mlh1-Mlh3 in the interference-dependent crossover pathway (see Introduction). Such a relationship might be expected if Mlh1-Mlh3 acts late in crossover resolution because a decrease in Mlh3 function would be expected to cause a random loss in crossing over, thus not assuring that all obligate crossovers would take place.

**Figure 4:**
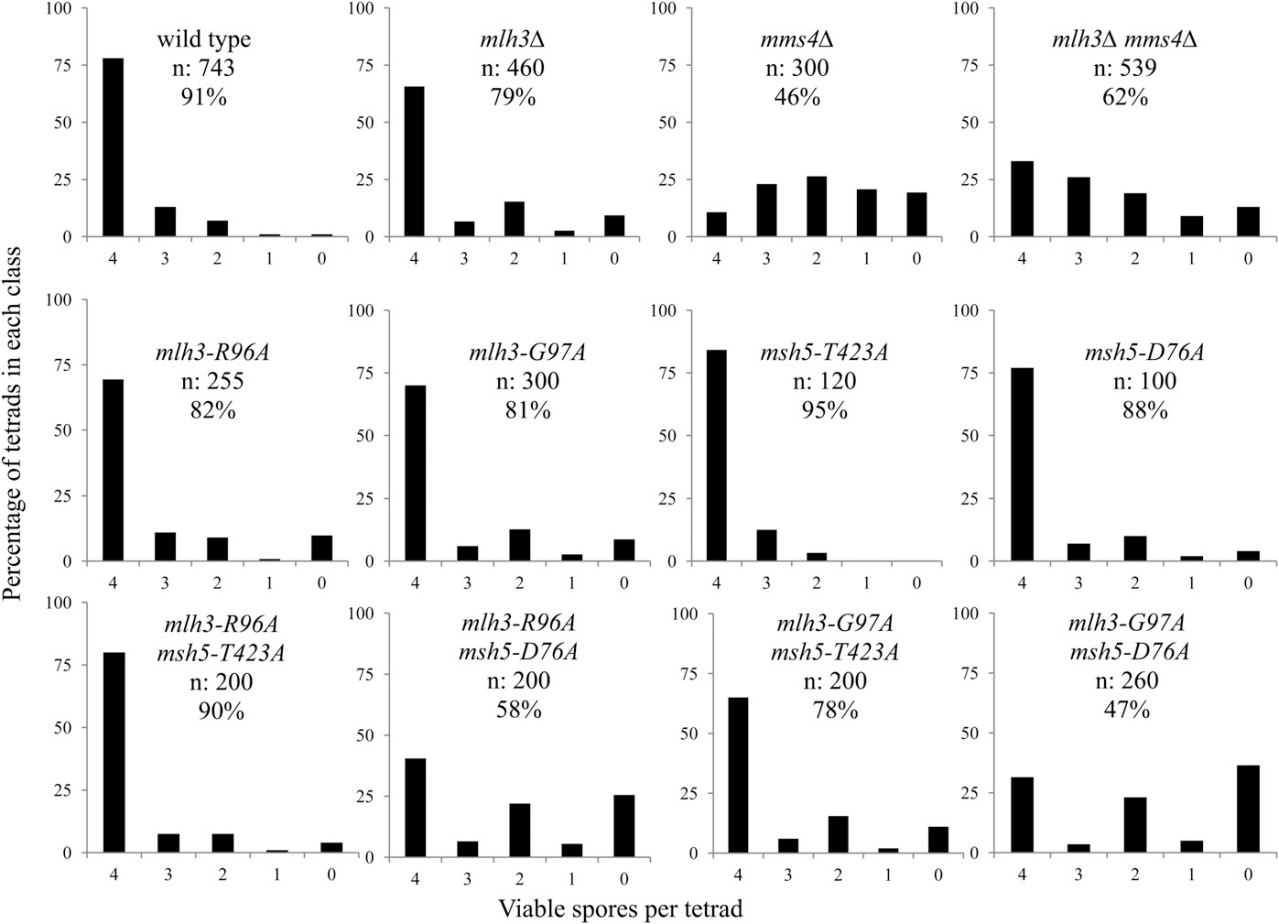
Spore viability profile of wild-type and select mutants. The horizontal axis shows the number of viable spores per tetrad, and the vertical axis shows the percentage of tetrads in each class. n, the total number of tetrads dissected, and percent spore viability are shown. Data for wild-type, *mlh3Δ*, *mms4Δ*, and *mlh3Δ mms4Δ* are from the NHY942/943 background (Tables 6 and 7; the remaining data are from the EAY1108/1112 background (Tables 4 and 5).

To further test whether the *mlh3* spore viability and map distance data support a roughly linear relationship, we more closely examined the phenotype of two mutants, *mlh3-G97A* and *mlh3-R96A*. These mutants show a relatively large difference in genetic map distance but a negligible difference in spore viability (*P* > 0.5). We attempted to detect any difference in phenotype conferred by these mutants by making double mutants with *msh5* alleles. When *mlh3-R96A* was combined with *msh5-T423A*, very little change in spore viability or map distance was observed compared with single mutants (Table 4; Figure 4). However, when the *mlh3-R96A* was combined with *msh5-D76A*, a strong synthetic defect was observed for spore viability in the double mutant; crossing over, however, was only slightly decreased. Similar results were obtained when each of these *msh5* alleles was combined with *mlh3-G97A*, except the results were more extreme. For example, the differences in spore viability between *mlh3-G97A msh5-D76A* and *mlh3-R96A msh5-D76A* (*P* < 0.02) and between *mlh3-G97A msh5-D423A* and *mlh3-R96A msh5-D423A* (*P* < 0.01) were statistically significant. This analysis confirms that *mlh3-G97A* confers a more severe defect compared with *mlh3-R96A*, as predicted if the pattern seen for *msh4/5* mutants did not hold for the *mlh3* mutants. Consistent with these observations, *mlh3-G97A* conferred a mild nondisjunction phenotype, as measured by an excess of 4, 2, 0 viable spore tetrads compared with 3 and 1 viable tetrads (Ross-Mcdonald and Roeder 1994), but *mlh3-G97A msh5-D76A* conferred a more extreme nondisjunction pattern (Figure 4).

### *mlh3Δ mms4Δ* mutants show dramatically decreased crossing over across four different chromosomes but display high spore viability

Our analysis of *mlh3* mutants described previously encouraged us to more closely examine *mlh3Δ* mutants for defects in crossing over. In previous studies authors showed that there are at least two types of crossover pathways in budding yeast: an Msh4-Msh5-Mlh1-Mlh3 pathway and an interference-independent pathway involving Mus81-Mms4 (see *Introduction*). In addition, three meiotic joint molecule resolvase complexes have been identified: Mus81-Mms4, Yen1, and Slx1-Slx4 (Boddy *et al.* 2001; Fricke and Brill 2003; Furukawa *et al.* 2003; Ishikawa *et al.* 2004; Cromie *et al.* 2006; Ip *et al.* 2008; Jessop and Lichten 2008; Oh *et al.* 2008; Muñoz *et al.* 2009; Svendsen *et al.* 2009; Schwartz and Heyer 2011). These resolvases appear to play different roles in different organisms. For example, Mus81-Mms4 plays a major role in fission yeast (Smith *et al.* 2003), but only a minor role in budding yeast, *Arabidopsis*, mouse, and *Drosophila* (De Los Santos *et al.* 2003; Argueso *et al.* 2004; Berchowitz *et al.* 2007; Trowbridge *et al.* 2007; Higgins *et al.* 2008; Holloway *et al.* 2008; Jessop and Lichten 2008; Oh *et al.* 2008).

Previously we showed that on a large chromosome, *mlh1Δ mms4Δ* double mutants display significant decreases (∼13- to 15-fold) in crossing over compared with wild type (Argueso *et al.* 2004). Based on these and other data we suggested that Mus81-Mms4 and Mlh1-Mlh3 act in competing crossover pathways (Argueso *et al.* 2004), with Mus81-Mms4 dependent crossovers promoting proper chromosome disjunction in the absence of Mlh1-Mlh3. Consistent with this finding, the Hunter lab and Lichten groups recently provided evidence for Msh4-Msh5-Mlh1-Mlh3-Exo1 and Mus81-Mms4 acting independently in crossover resolution (De Muyt *et al.* 2012;Zakharyevich *et al.* 2012). The Hunter lab previously showed that *mlh3Δ* decreases crossover levels without changing joint molecule levels, also suggesting a late role for Mlh3 (Zakharyevich *et al.* 2010). Using Southern blot analysis at the well-studied *HIS4LEU2* hotspot, they showed that compared with the wild-type, *exo1* (Exo1 forms a complex with Mlh1-Mlh3) reduced crossing over by 49%, *mms4yen1* by 39%, and *exo1mms4yen1* by 86%. Strikingly, crossover levels decreased roughly 20-fold in *mlh3mms4slx4yen1sgs1* cells (Zakharyevich *et al.* 2012). The Lichten group (De Muyt *et al.* 2012) showed that in *msh4Δ mms4yen1Δ* triple mutants, the bulk of chromosomal DNA fails to segregate. Furthermore, they found that unresolved joint molecules accumulated to similar levels in *msh4Δ ndt80Δ*, where joint molecule resolution cannot take place, suggesting that the Mus81-Mms4 and Yen1 pathways are responsible for resolving crossover intermediates that are not resolved by the Msh4-Msh5-Mlh1-Mlh3 pathway. Because they found that most joint molecules were resolved in *mms4yen1Δ slx1Δ* mutants, their data provide evidence that Msh4-Msh5-Mlh1-Mlh3 acts in crossover resolution.

The Hunter and Lichten studies, summarized previously, provide evidence that Exo1-Mlh1-Mlh3 and Mus81-Mms4 are responsible for the majority of crossovers in budding yeast. Although each of the aforementioned studies presented convincing data for the presence of two independent crossover pathways, physical data reported in Zakharyevich *et al.* (2012) were primarily obtained at a single locus, the *HIS4LEU2* hotspot, and genetic data were obtained by Argueso *et al.* (2004) and Nishant *et al.* (2008) in only one chromosome arm. To understand the role of Mlh3 in crossing over genome-wide, we analyzed spore viability and crossovers across four chromosomes in *mlh3Δ mms4Δ* double mutants. A total of 250 cM of map distance was measured, representing ∼6.2% of the yeast genome. *mlh3Δ mms4Δ* double mutants were chosen for this analysis because they formed viable spores at a reasonable frequency and displayed strong defects in crossing over in one arm of chromosome XV. As shown in Tables 6 and 7 and Figure 2, we found that for all loci examined crossing over was drastically reduced (6- to 17-fold) in *mlh3Δ mms4Δ* strains compared to wild-type. Interestingly, crossing over was decreased by the smallest amount on chromosome III, a pattern seen in other meiotic mutants (Zanders and Alani 2009). Although *mlh3Δ* mutants show a characteristic 4:2:0 pattern of viable spores per tetrad indicative of nondisjunction (Ross-Macdonald and Roeder 1994; Hollingsworth *et al.* 1995; Hunter and Borts 1997; Argueso *et al.* 2003; Nishant *et al.* 2008; this study), neither *mms4Δ* nor *mlh3Δ mms4Δ* showed this pattern (Figure 4). Thus, our analysis provides further support for the hypothesis that Mlh1-Mlh3 and Mus81-Mms4 independently contribute late roles for meiotic crossover formation.

**Table 6:** Spore viabilities and cumulative genetic map distances for wild type, *mlh3Δ*, *mms4Δ*, and *mlh3Δ mms4Δ* for chromosomes III, VII, VIII, and XV

Genotype			Map Distance, cM			
Chromosome	Spore Viability, %	n	III (333 kb)	VII (1040 kb)	VIII (582 kb)	XV (1095 kb)
Wild type*^a^*	91.0	572	34.9	68.7	46.2	96.1*^b^*
*mlh3Δ*	79.0	306	29.3	32.4	20.3	54.5*^c^*
*mms4Δ*	46.3	32	32.7	50.0	31.8	83.4*^b^*
*mms4Δ**^d^*	45.4	272	25.2	62.1	35.3	
*mlh3Δ mms4Δ*	61.9	170	5.7	9.6	2.8	8.4*^c^*
Fold decrease in *mlh3Δ mms4Δ vs.* wild type			6.1	7.2	16.5	11.4

Spore viabilities (%) and cumulative genetic map distances in cM (number of complete tetrads) on chromosomes III, VII, VIII, and XV are shown for *mlh3Δ*, *msh5Δ*, *mlh3* alleles, *msh5* alleles, and the double mutants (Tables 1 and 2). Sizes of each chromosome are shown below each chromosome number, and the fold decrease in crossing over in *mlh3Δ mms4Δ* compared with wild type is shown below. Chromosome III, VII, and VIII data are from derivatives of the isogenic SK1 NHY942/943 background. Data for chromosome XV are from derivatives of the congenic SK1 EAY1108/1112 background.

a Data from Zanders and Alani (2009).

b Data from Argueso *et al.* (2004).

c Data from Nishant *et al.* (2008).

d Data from De Los Santos *et al.* (2003).

**Table 7:** Genetic map distances for chromosomes III, VII, and VIII from single spores and tetrads with distributions of recombinant and parental progeny

	Single Spores				Tetrads				
Genotype	n	Par.	Rec.	cM	n	PD	TT	NPD	cM
Chromosome III									
* HIS4-LEU2*									
Wild type*^a^*	2711	2360	351	12.9	572	413	141	2	12.6-15.0
* mlh3Δ*	1453	1333	120	8.3	306	253	47	1	7.4-10.3
* mms4Δ*	555	508	47	8.5	32	21	5	0	5.8-13.5
* mlh3Δ mms4Δ*	1336	1304	32	2.4	170	158	2	0	0.2-1.1
* LEU2-CEN3*									
Wild type*^a^*	2711	2527	184	6.8	572	488	68	0	5.4-6.8
* mlh3Δ*	1453	1314	139	9.6	306	261	39	1	6.1-8.9
* mms4Δ*	555	482	73	13.2	32	22	3	1	5.8-28.8
* mlh3Δ mms4Δ*	1336	1302	34	2.5	170	156	4	0	0.6-1.9
* CEN3-MAT*									
Wild type*^a^*	2711	2309	402	14.8	572	395	160	1	13.9-15.9
* mlh3Δ*	1453	1246	207	14.2	306	223	78	0	11.7-14.2
* mms4Δ*	555	464	91	16.4	32	23	3	0	2.6-8.9
* mlh3Δ mms4Δ*	1336	1288	48	8.5	170	153	6	1	1.8-5.8
Chromosome VII									
* TRP5-CYH2*									
Wild type*^a^*	2711	1803	908	33.5	572	197	337	9	34.2-37.8
* mlh3Δ*	1453	1215	238	16.4	306	198	100	0	15.4-18.2
* mms4Δ*	555	391	164	29.5	32	11	11	0	19.7-30.3
* mlh3Δ mms4Δ*	1336	1289	47	3.5	170	151	11	0	2.4-4.4
* CYH2-MET1:*									
Wild type*^a^*	2711	2451	260	9.6	572	442	101	0	8.5-10.1
* mlh3Δ*	1453	1350	103	7.1	306	266	32	0	4.5-6.3
* mms4Δ*	555	500	55	9.9	32	18	4	0	5.0-13.2
* mlh3Δ mms4Δ*	1336	1302	34	2.5	170	156	6	0	1.1-3.0
* MET13-LYS5:*									
Wild type*^a^*	2711	2152	559	20.6	572	334	205	4	19.6-22.6
* mlh3Δ*	1453	1307	146	10.0	306	242	55	1	8.7-11.7
* mms4Δ*	555	461	94	16.9	32	15	7	0	10.9-20.9
* mlh3Δ mms4Δ*	1336	1271	65	4.9	170	148	14	0	3.2-5.4
Chromosome VIII									
* CEN8-THR1:*									
Wild type*^a^*	2711	2105	606	22.4	572	317	219	2	20.2-22.8
* mlh3Δ*	1453	1305	148	10.2	306	251	45	0	6.6-8.6
* mms4Δ*	555	463	92	16.6	32	16	6	0	8.9-18.4
* mlh3Δ mms4Δ*	1336	1288	48	3.6	170	157	3	0	0.4-1.5
* THR1-CUP1:*									
Wild type*^a^*	2711	2043	668	24.6	572	277	260	1	23.5-25.9
* mlh3Δ*	1453	1258	195	13.4	306	226	69	1	11.1-14.2
* mms4Δ*	555	427	128	23.1	32	14	8	0	13.1-23.3
* mlh3Δ mms4Δ*	1336	1292	44	3.3	170	154	6	0	1.1-2.6

Strains analyzed are isogenic derivatives of the SK1 NHY942/943 background (Tables 1 and 2). Single spore data are shown with n, total number of spores, and parental and recombinant data. Map distances (cM) were calculated by recombination frequency (recombinant spores/total spores) × 100. Tetrad data are shown with n, number of complete tetrads. Map distances (cM) were calculated using the Perkins formula (Perkins 1949), and 95% confidence intervals were calculated using the Stahl Laboratory Online Tools website (http://www.molbio.uoregon.edu/∼fstahl/).

a Data from Zanders and Alani (2009).

Previous work showed that *mms4Δ* strains display low spore efficiency (∼10%) and viability (∼40%) as well as high levels of aberrant recombination events (De Los Santos *et al.* 2001, 2003). We found that the *mlh3Δ* mutation can partially suppress the spore viability, sporulation defects, and high frequency of aberrant events observed in *mms4Δ* strains (Tables 6 and 8). In the SK1 isogenic background NHY942/943, *mms4Δ* strains displayed low sporulation efficiency (16%) and viability (45%) whereas *mlh3Δ* displayed greater levels of spore formation (73%, *P* < 0.001) and viability (79%, *P* < 0.001). *mlh3Δ mms4Δ* displayed significantly greater sporulation (43%; *P* < 0.001) and viability (62%; *P* < 0.001) compared to *mms4Δ*. In addition, *mlh3Δ mms4Δ* mutants showed gene conversion levels that were similar to wild-type but lower than *mms4Δ* alone (Table 8; aberrant levels for our small *mms4Δ* data set are similar to those seen in De Los Santos *et al.* (2003), who analyzed 272 tetrads).

**Table 8:** Aberrant marker segregation in wild type, *mlh3Δ*, *mms4Δ*, and *mlh3Δ mms4Δ* on chromosomes III, VII, and VIII

Chromosome III	Four-spore viable tetrads	*HIS4*	*LEU2*	*ADE2*	*MATa*	Total
Wild type	572	2.1	0.3	0.2	0.2	2.8
* mlh3Δ*	306	0.7	0.7	0.3	0.0	1.7
* mms4Δ*	32	9.4	6.3	3.1	3.1	21.9
* mlh3Δ mms4Δ*	170	4.1	0.6	0	1.2	5.9
Chromosome VII		*LYS5*	*MET13*	*CYH2*	*TRP5*	
* Wild type*	572	1.6	2.4	0.3	0.7	5.0
* mlh3Δ*	306	0.7	2.0	0.0	0.0	2.7
* mms4Δ*	32	9.4	0.0	6.3	0.0	15.7
* mlh3Δ mms4Δ*	170	1.2	2.4	0.0	1.2	4.8
Chromosome VIII		*URA3*	*THR1*	*CUP1*		
Wild type	572	0.2	5.1	0.7		6.0
* mlh3Δ*	306	0.0	3.3	0.0		3.3
* mms4Δ*	32	0.0	6.3	9.4		15.7
* mlh3Δ mms4Δ*	170	0.6	4.7	0.6		5.9

Aberrant segregation (1:3 or 3:1) of markers is shown. Data are from four-spore viable tetrads analyzed by RANA software (Argueso *et al.* 2004). Strains analyzed are isogenic derivatives of the SK1 NHY942/943 background (Tables 1 and 2).

**Figure 5:**
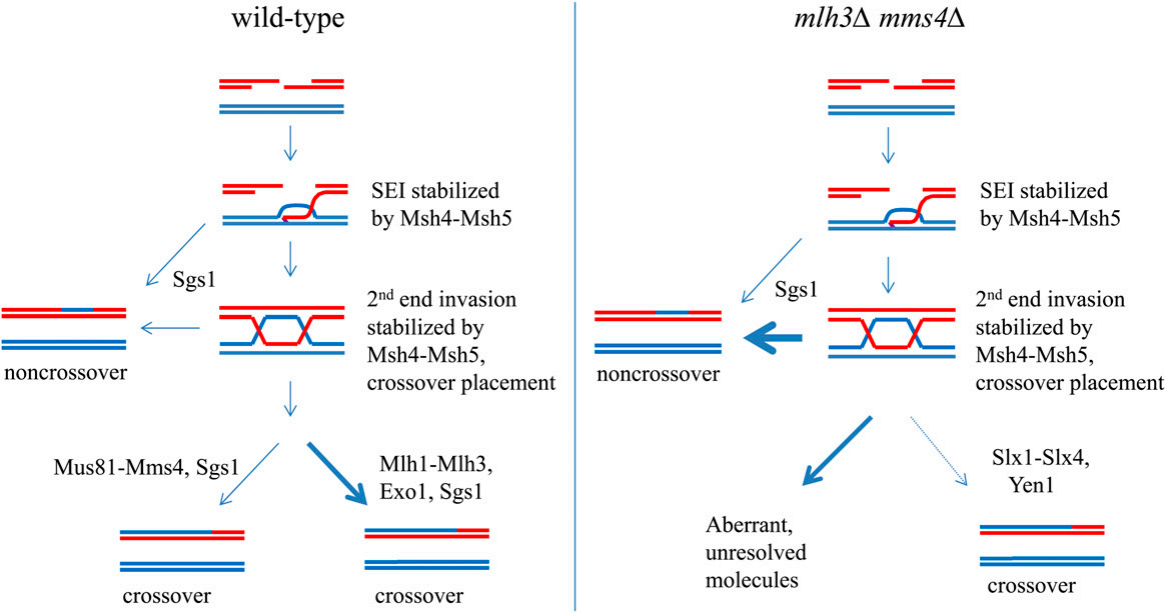
Model of crossover pathways during meiosis. A summary of the crossover pathways are shown. In wild-type cells (left), DSBs are made and resected, and initial single-end invasion intermediates can be dissolved by Sgs1−dependent mechanisms, leading to noncrossovers. Single-end invasion intermediates that are not resolved as noncrossovers can proceed through the Mus81-Mms4 interference-independent pathway, leading to crossovers, or Msh4-Msh5 can stabilize the SEI in an interference-dependent mechanism. These stabilized joint molecules undergo crossover placement decisions, and are subsequently resolved in an Mlh1-Mlh3-dependent manner. In the absence of Mlh3 and Mms4 (right), initial recombination events occur as in wild type. However, due to the lack of the major Mlh1-Mlh3 and Mus81-Mms4 resolvase functions, other pathways are activated, including Sgs1-dependent resolution to form noncrossovers and other resolution activities (*e.g.*, Slx-Slx4, Yen1), resulting in a larger number of events being resolved into noncrossovers.

Our measurements of gene conversion in *mlh3Δ mms4Δ* mutants, coupled with previous analyses of recombination intermediates in crossover resolution mutants, are consistent with meiotically induced DSBs forming at wild-type levels in *mlh3Δ mms4Δ* strains [Table 8 (Argueso *et al.* 2004; Nishant *et al.* 2010; Zakharyevich *et al.* 2012). Based on this argument, we are left trying to understand how recombination intermediates in *mlh3Δ mms4Δ* are repaired. Previous genetic and physical studies have identified roles for Sgs1 in resolving aberrant joint molecules that form during meiosis in mutants defective in Mus81-Mms4 and Mlh1-Mlh3 crossover pathways (Van Brabant *et al.* 2000; Adams *et al.* 2003; Rockmill *et al.* 2003; Wu and Hickson 2003; McVey *et al.* 2004; Bachrati *et al.* 2006; Jessop *et al.* 2006; Oh *et al.* 2007, 2008; Cejka and Kowalczykowski 2010; De Muyt *et al.* 2012; Zakharyevich *et al.* 2012). Based on the aforementioned studies we hypothesize that Sgs1 is acting to resolve joint molecules into noncrossovers in *mlh3Δ mms4Δ* mutants (Figure 5). One explanation for why the spore viability of *mms4Δ* is lower than that seen in *mlh3Δ mms4Δ* is that in *mms4Δ* mutants Mlh1-Mlh3 competes with Sgs1 for joint molecule substrates but is unable to efficiently resolve them. The explanation is consistent with chromosome segregation defects seen in *mms4* mutants and the finding that *sgs1mms4* mutants accumulate high levels of joint molecules in meiosis (Oh *et al.* 2008).

### Chromosome disjunction appears mostly functional in *mlh3Δ mms4Δ* despite dramatic genome-wide decreases in crossing over

As indicated previously, spore viability in *mlh3Δ mms4Δ* is high (62%) despite large reductions (6- to 17-fold) in crossing over. Such reduced levels should yield crossover levels below the obligate number (16) required to segregate all yeast homologs. If we assume that crossover levels decrease to similar extents across the length of a single chromosome, then only chromosome VII would appear to have at least one crossover in *mlh3Δ mms4Δ*. This calculation is based on high-resolution genotyping of meiotic spore progeny performed by Mancera *et al.* (2008). They observed in wild-type an average of three, eight, four, and seven crossovers on chromosomes III, VII, VIII, and XV, respectively. Based on these values, multiple chromosomes are unlikely to receive a crossover during meiosis in *mlh3Δ mms4Δ*.

We offer two explanations for the high spore viability in *mlh3Δ mms4Δ*, both of which assume achiasmate chromosome disjunction mechanisms. The first suggests that the high spore viability is due to distributive disjunction, which is defined as the process in which “two nonhomologous chromosomes that lack homologs or two homologs that have failed to recombine, disjoin at meiosis I” (Guacci and Kaback 1991). Distributive disjunction has been shown to accurately segregate chromosomes in male *Drosophila* meiosis and the fourth chromosome in female *Drosophila* meiosis (Grell 1962, 1976). It also plays a role in budding yeast (Guacci and Kaback 1991; Loidl *et al.* 1994). However, distributive disjunction in budding yeast acts independently of chromosome homology and chromosome size, at least when only three achiasmate elements are present (Guacci and Kaback 1991; Loidl *et al.* 1994; Ross *et al.* 1996). Based on this observation, it is unlikely that such a system would efficiently act to segregate chromosomes in meiosis I if multiple chromosomes lacked chiasma. Indeed, hybrid yeast strains that have severely reduce recombination due to high sequence divergence display low spore viability (∼1%; Hawthorne and Philippsen 1994; Hunter *et al.* 1996).

A second explanation is that homologous pairing mechanisms are taking place in *mlh3Δ mms4Δ* that promote disjunction of homologs in the absence of crossing over. We can imagine two ways that this could happen: (1) Chromosome disjunction in *mlh3Δ mms4Δ* is facilitated by Zip1, a synaptonemal complex protein that promotes homology-independent centromere pairing (Tsubouchi and Roeder 2005; Gladstone *et al.* 2009; Newnham *et al.* 2010). Zip1 promotes centromere pairing in both nonhomologous chromosomes and nonexchange homologous chromosomes, providing a mechanism for nonexchange chromosomes to be held together until the first meiotic division, possibly by promoting correct spindle orientation (Newnham *et al.* 2010; Gladstone *et al.* 2009). (2) Msh4-Msh5 acts to facilitate disjunction in *mlh3Δ mms4Δ* by promoting homolog pairing. Consistent with this idea, Msh5 has been shown to act in early steps in homolog pairing in mice and *Sordaria* (Edelmann *et al.* 1999; Storlazzi *et al.* 2010). Experiments aimed at testing these ideas are in progress.
